# An agricultural survey for more than 9,500 African households

**DOI:** 10.1038/sdata.2016.20

**Published:** 2016-05-24

**Authors:** Katharina Waha, Birgit Zipf, Pradeep Kurukulasuriya, Rashid M. Hassan

**Affiliations:** 1 CSIRO Agriculture, 306 Carmody Road, St Lucia, QLD 4067, Australia; 2 University of Potsdam, Institute of Earth and Environmental Science, Karl-Liebknecht-Strasse 24-25, Potsdam 14476, Germany; 3 Centre for Environmental Economics and Policy Analysis in Africa (CEEPA), Faculty of Natural and Agricultural Sciences, University of Pretoria, Pretoria 0001, Republic of South Africa; 4 †Unaffiliated: pkurukulasuriya@gmail.com

**Keywords:** Agriculture, Social sciences

## Abstract

Surveys for more than 9,500 households were conducted in the growing seasons 2002/2003 or 2003/2004 in eleven African countries: Burkina Faso, Cameroon, Ghana, Niger and Senegal in western Africa; Egypt in northern Africa; Ethiopia and Kenya in eastern Africa; South Africa, Zambia and Zimbabwe in southern Africa. Households were chosen randomly in districts that are representative for key agro-climatic zones and farming systems. The data set specifies farming systems characteristics that can help inform about the importance of each system for a country’s agricultural production and its ability to cope with short- and long-term climate changes or extreme weather events. Further it informs about the location of smallholders and vulnerable systems and permits benchmarking agricultural systems characteristics.

## Background & Summary

Agriculture is one of the most important economic sectors in many African countries and provides livelihoods for large parts of the population. As there are many smallholder farmers and fields are heterogenic and scattered with a large variety of crops grown and dynamic interactions between crops and livestock many characteristics of African households in rural areas are not well known or uncertain. Facing challenges such as global climate change, population growth and urbanization, changing dietary patterns and increased pressure on the land and water resources it is crucial to understand characteristics and dynamics of rural households. Agricultural household surveys can provide a meaningful data base for measuring the performance of households, farming and management practices, vulnerability to climate change and risk management strategies. Among the existing and published African household surveys known to the authors^1–4^ the household survey presented here is the most comprehensive survey in terms of number of households interviewed and geographic coverage. The original purpose of the data set was to develop methods to assess how climate affects current agricultural practices in Africa, analyse how these practices might be affected by future climate change including adaptation choices that are made in the future. Household surveys were conducted in the growing seasons 2002/2003 or 2003/2004 in eleven African countries: Burkina Faso, Cameroon, Ghana, Niger and Senegal in western Africa; Egypt in northern Africa; Ethiopia and Kenya in eastern Africa; South Africa, Zambia and Zimbabwe in southern Africa. Study regions were chosen to cover all key agro-climatic zones and farming systems in Africa.

This dataset is the product of the World Bank/Global Environmental Facility project *Climate, Water and Agriculture: Impacts on and Adaptations of Agro-ecological Systems in Africa* that was coordinated by the Centre for Environmental Economics and Policy for Africa (CEEPA) at the University of Pretoria, South Africa in association with Yale University, USA. The research was undertaken by a number of economists, agronomists and climate scientists from the participating countries as well as with support from staff from World Bank, FAO, the International Water Management Institute, Yale University and University College London.

The data was already used by many researchers for various purposes and produced a very large body of research reports, journal articles, chapters and books and a number of PhD theses. First the questionnaire was published in a book^5^ and the data set was used to describe the likely impacts of climate change on agriculture in Africa. This analysis largely focused on modelling how net revenue per hectare for smallholder and commercial farms in Africa would be affected by climate change. The analysis drew on and built upon the Ricardian method of analysing climate change impacts on the agriculture sector^6^ where cross-sectoral data from across all eleven countries was studied. In parallel each country team used its respective country dataset to carry out country level analyses on various aspects of climate change impacts and adaptation to climate change among African farmers. Country level data were then aggregated to carry out analyses at the continent level. Summaries of these reports are provided as discussion papers in the list of policy notes (No. 8 to 39) produced by CEEPA^7^. The database had also supported the completion of a number of PhD theses at the University of Pretoria and Yale University. Until now 19 journal articles and policy notes have been published using this dataset over the last ten years. So far they focused on irrigation^8^, crop selection^9,10^, economic impacts of climate change on agriculture^11–16^, livestock^17^ adaptation and vulnerability^18–26^ and farming and management practices in Africa^27^. Further to these studies we envision that many data generated from the survey can be used to characterise and benchmark farming practices of mostly smallholders but also larger agricultural enterprises.

## Methods

### Basic characteristics and geographic coverage

**Figure 1:**
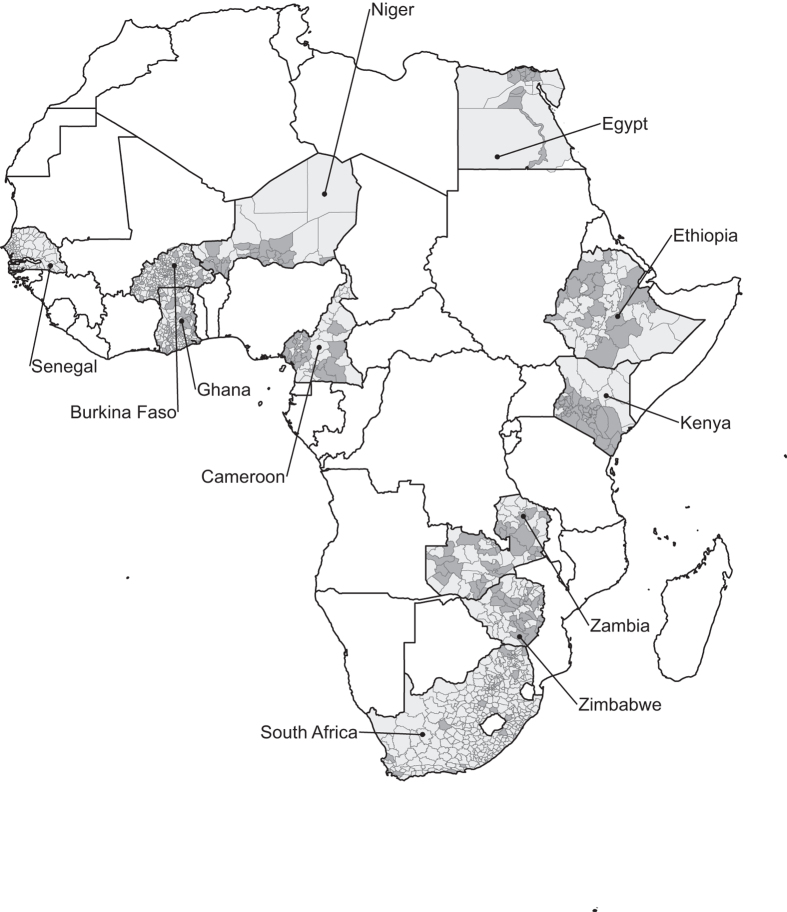
Spatial coverage of districts (AU2) in the survey. Districts in the survey are shown in dark grey and all districts in a surveyed country are shown in light grey.

Basic information for each household is a household ID, the location of the farm (country, region, district, subdivision, and village), the name of the interviewer, the time required for the interview, the type of farm entity e.g., small-scale or large-scale and the relationship of the respondent to the head of the household. The total number of households in the data set is 9,597. Households were chosen randomly within districts representing the different agro-ecological zones in a country. Most of the surveys are for the 2002–2003 agricultural year, collected in 2003–2004. Data from Cameroon, Ethiopia, Kenya and Zimbabwe are for the 2003–2004 agricultural year, collected in 2004–2005. Table 1 shows the number of regions, districts, subdivisions and households per country and Fig. 1 shows the spatial coverage of the surveyed districts.

### Questionnaire

The questionnaire is organised into seven sections and respondents were asked to relate the information provided to the previous 12 months’ farming season:

- Household roster
- Members of households (gender, age, education, tribe, religion)
- Household size
- Work on farm activities and/or non-farm activities
- Access to electricity
- Employment
- Occupation and time spent
- Time lost due to illness
- Tenure Issues and labour composition
- Number and size of separated farm area
- Farming System
- Tenure type, rent paid, number of years used plots
- Value of farm
- Labour information per season e.g., land preparation, planting, weeding
- Wage for hired workers
- Details on farm activities Part I: Food and tree crops (56 crop types)
- Type, planting date, harvest date, proportion of area cultivated, quantity harvested
- Amount consumed, sold, lost, value of crops sold, amount of seeds
- Average yield
- Water source
- Fertilizer use, pesticide use
- Farm machinery, equipment and farm buildings
- Distance to market for selling and buying inputs, form of transport to market
- Total costs of transport, marketing, storage, post-harvest loss
- Details on farm activities Part II: Livestock, poultry and other farm animals (8 types of animals, 7 types of products)
- Number of animals owned, born, lost, stolen, killed, purchased
- Number of months that animals graze on
- Purchase prize, sale prize
- Quantity of products for own use and sold
- Total costs of transport, marketing, storage, post-harvest loss
- Access and extension services
- Frequency of advice from extension workers
- Organization and payment for extension service
- Information on expected rainfall provided
- Other farming costs and farm subsidies
- Total household net income
- Percentage of income from non-farm activities and non-agriculture activities
- Taxes paid
- Interest rate for lending money
- Subsidies received
- Adaptation options
- Number of years of being a farmer
- Adaptation strategies to climatic variation and main causes
- Any long-term shifts noticed in temperature and precipitation and adjustments

### Key household and farm characteristics

In over 70% of the households the head of the household was the respondent. Half of the households are small-scale farmers, the other half are medium- or large-scale farmers. Each farm type was surveyed in each country and with the exception of Kenya, Senegal and South Africa more than 80% of the farms were small or medium-scale farms. In Ghana, Zambia and Zimbabwe more than 80% of the households were smallholders. In contrast, 73% of all households in Senegal belong to a large-scale farm. Defining the farm size is country-specific and depends on the self-assessment of the households. For example, the average farmed land area of small farms in Egypt is approximately 0.7 ha, whilst it reaches 51 ha in South Africa. 50% of the households in Egypt have 0.5 ha or less farmed land area available while in Niger they have 5.5 ha or less farmed land area. The majority of households received less than 20% of their total household net income from non-farm activities in Burkina Faso, Cameroon and Egypt, while income from non-farm activities is more important in the other countries e.g., in Zambia about 35% of all households receive more than 80% of their income from non-farm activities.

### Crop and livestock types and products

The majority of households grew at least one crop on one plot in a season and continuous cropping with or without a fallow period is the most common farming system in most of the countries except for South Africa and Kenya where livestock farming dominates. About 350 households did not grow any crops in the surveyed agricultural season.

The household survey reports cropping activities for 56 different crops and tree crops which are grown on up to three plots in up to three seasons within 12 months. Some households grew up to six crops simultaneously on a plot. The crop types included in the survey are alfalfa, banana, barley, beans, cashew, cassava, citrus fruit, chickpeas, clover, cocoa, cocoyam, cowpea, coffee, cotton, cucumber, enset, field pea, flax, garden-eggs, garlic, grape, groundnut, kola, lentil, mango, maize, millet, oil palm, okra, onion, palm dates, paprika, peanuts, pepper, pigeon pea, pineapple, plantain, potato, rice, safflower, sesame, shallots, sheanut, sorghum, soybean, spinach, squash, sugar cane, sunflower, tea, tef, tobacco, tomato, wheat, and yam. There is also a group of other crops. The ten most frequently grown crops across all eleven countries are, in descending order, maize, millet, groundnut, sorghum, beans, cowpea, cassava, wheat and cotton. The group ‘other crops’ ranks fifth in this list occurring more frequently than beans, indicating a large diversity of crops in this group.

More than 5,000 farmers are livestock farmers. The livestock data identifies the five major types of livestock in Africa as beef cattle, dairy cattle, goats, sheep, and chickens. Other less frequently recorded animals include pigs, breeding bulls, oxen, camels, ducks, guinea fowl, horses, bees, and doves. The major livestock products sold were milk, meat, eggs, wool, and leather^28^.

## Data Records

The data set language is English and the geographic location is Africa (34° S to 37°N and 25°W to 47°E). The data of this resource (Data Citation 1) are freely available through figshare (http://figshare.com/), under the CC0 waiver. We confirm that we have appropriate approval to share these data under this waiver. Data can be downloaded in one archive file (CEEPA.zip) which contains the responses of each household head to each question in the survey either as a STATA file (CEEPASurvey.dta) or a tab-delimited TXT file (CEEPASurvey.txt). These files contain the main data set organized in 9,597 rows, each corresponding to a household and 1,753 columns, each corresponding to the response to an answer in the questionnaire. The dta file can be read with STATA or with R using read.dta() {foreign}. The archive file also contains several metadata that describe the structure of the data set:

- Questionnaire.pdf: This file contains the questionnaire used, a description for each variable name and the question ID.
- SurveyManual.pdf: This file gives further information on the household questionnaire, the research design and surveying. It was produced for the team leaders and interviewers in the World Bank/GEF project.
- AdaptationCoding.pdf: This file describes codes for variables ‘ad711’ to ‘ad7625’ from section VII of the questionnaire on adaptation options.

### Mapping the data

The administrative unit of each household can be mapped using the information in Supplementary File 1. This file helps assigning the district names used in the survey (variable ‘adm2’) to the district names in shape files from the GADM data base of Global Administrative Areas (http://www.gadm.org/) for mapping the data. The shape files can be extracted for each country separately from the GADM database, version 2.0, December 2011. The names of shapefiles in column ‘Name.GADM’ are names of administrative units levels 2 for all countries except Egypt (level 1) and Senegal (level 3).

## Technical Validation

Multi-stage stratified random sampling was employed to select sample households to be surveyed. In the first stage eleven countries were selected to represent the four sub-regions of Africa East, West, North and Southern Africa. Countries from each sub-region were selected based on formal expression of interest from respective institutions within countries concerned with managing climate change impacts. In the second stage districts were selected to capture representative farms across diverse agro-climatic conditions within each country according to the FAO classification of agro-ecological zones and farming systems. Stage three sampling involved selection of villages within districts included in the survey. In each district, surveys were conducted of farms randomly selected from a list of farmers prepared with the assistance of respective district level agricultural authorities. Sampling was clustered in villages to reduce the cost of administering the survey.

### Reducing sampling errors

A number of procedures in the sampling design aim at ensuring reliable and unbiased data production. During a project meeting of the country teams the sampling technique was defined to minimize sampling errors that would arise from choosing an inappropriate sample size or representation of different groups. The number of sampling units (preferred 2nd level administrative unit such as districts) should be between 30 and 60. Agriculture should be a major activity in the chosen units. Within each sampling unit a minimum of two farm types i.e. large and small and a maximum of five farm types, also considering other farming characteristics as farm size are to be surveyed. A sample with each farm type should consist of 5–10 randomly selected households and commercial farms engaged in agriculture during the last 12 months. The total sample size in each country should be between 800 and 1,000 households.

The interviewers were conducted as face-to-face interviews with the advantage of a high response rate and being able to ask complex questions, clarify questions and control their sequence. On the other hand, face-to-face interviews are costly and time consuming, answers might be filtered or the interviewer’s presence affect the response in any other way^29^.

The questionnaire includes open and closed questions. Closed questions aim at classifying households and farms by asking for e.g., age, gender (categorical data), income, farm size (ranked data), number of household members, production quantity (scored data) and at exploring farmers behaviors by asking for e.g., the number of crops grown, fertilizer and pesticide use or the division of labor. The questionnaire gives clear instructions on answering each question by giving predefined answers or categories and options such as ‘other’. Open questions are only used in section VII: Adaptation options. Farmers were asked about their experience and perception of shifts in climate, their main adaptation strategies and constraints for making adjustments. Instructions for interviewers such as ‘Please allow for multiple responses’, ‘Please denote the unit in which the quantity of land is measured locally during interview’ and clarifications such as ‘One day of work 6–8 h of work’ in the questionnaire aim at minimizing sampling errors. A 12 pages manual (Data Citation 1) provides additional instructions for interviewers.

### Reducing non-sampling errors

Even though household surveys are widely used tools not much is known about the magnitude of non-sampling errors in surveys in developing countries and their impact^30^. Phung *et al.*^30^ highlight two main reasons for this: a missing consistent procedure on how to deal with common issues such as outliers and missing data and missing information on the causes of errors. The most important types of non-sampling errors are non-response errors and measurement errors. They can occur because interviewers deviate from the established study design, influence the respondent or the respondent cannot remember details e.g. on labour distribution among different household members^30^ or on details for time periods in the past. Reserve lists were used to manage non-response and replace selected respondents who were unavailable or unable to provide required information at the time of survey. Multiple visit surveys to reduce memory and recall biases were not feasible considering cost implications of such wide geographic spread of the sample. In the following and in Supplementary File 2 we give examples of such non-sampling errors and other uncertainties in the data set.

The name of the interviewer was reported for Burkina Faso, Ethiopia, Egypt, Ghana, Kenya Niger, Senegal, South Africa and Zambia so is missing only for Cameroon and Zimbabwe. Names of interviewers were anonymized by using numeric codes. The date of the interview was only recorded for Zimbabwe. The name of the level three administrative unit and/or the name of the village was not reported except for Kenya, Burkina Faso, Zambia and Senegal. For Cameroon, there is no data on adaptation options. In some previous studies the authors decided to not use the data e.g., for Zimbabwe^28^ because of the turbulent economic and political situation in that country during the survey. Overall there is information missing for 7% of all variables and 58% of all the data entries were NAs. NAs indicate that respondents did not know the answer, did not want to answer the questions but also that the question did not apply to them as questions in section III and IVa and IVb asking for the division of labour or farming activities in a second or third growing season. About 37% of all households had NAs for half of all data entries.

The data on fertilizer use, pesticide use and irrigated area was compared to national average values from the FAO and the World Bank^27^. Mean fertilizer consumption per unit area as reported in the household survey often exceeds country average fertilizer consumption rates as reported from FAO and World Bank which has two main reasons: first, households most likely reported on traditional, organic fertilizers like plant residue and animal manure in contrast to inorganic fertilizer rates reported from the FAO and World Bank, and second, the area that the FAO and the World Bank relate their data to is defined as total arable land including fallow land and temporary meadows for moving and pasture so its larger than the plot sizes reported in the survey. In several countries the median fertilizer consumption is closer to the official national statistics than the means and the fact that the 0.1 and 0.25 quantiles are zero or close to zero indicates that many households do not apply fertilizer at all but a small number of households have a high fertilizer consumption. Pesticide consumption as reported in the household survey is also above FAO data which might reflect the inclusion of traditional biological pesticides not included in the national average statistics^27^.

## Usage Notes

This data set can be used to investigate the characteristics of agricultural systems in Africa at one point in time but across spatial dimensions. It may help to improve understanding decision-making of African farmers including risk management strategies in different agro-ecological zones in low- and middle-income countries. We emphasize that the results of such analysis should be interpreted within the context of the dataset considering its bias and limitations described in this paper. Preferably results should be cross-checked with results from other household surveys.

## Additional Information

**How to cite this article:** Waha, K. *et al.* An agricultural survey for more than 9,500 African households. *Sci. Data* 3:160020 doi:10.1038/sdata.2016.20 (2016).

## Supplementary Material



Supplementary File 1

Supplementary File 2

## Figures and Tables

**Table 1:** Basic information on geographic coverage of CEEPA household survey.

**Country**	**Number of administrative units level 1 (AU1)**	**Number of administrative units level 2 (AU2)**	**Number of villages in AU2**	**Number of households**	**Ø households per AU1**
Burkina Faso	42	48	40	1,087	26
Cameroon	10	30	—	800	80
Egypt	3	20	—	900	300
Ethiopia	7	32	—	988	143
Ghana	13	61	—	894	69
Kenya	11	44	573	816	74
Niger	6	30	—	900	150
Senegal	9	69	219	1,078	120
South Africa	9	17	—	416	46
Zambia	9	30	529	1,008	112
Zimbabwe	6	24	—	700	117
Administrative units level 2 are mostly referred to as districts in many countries, so we continue using this terminology in the paper.					
