# Supplementary material for: An agricultural survey for more than 9,500 African households
Source: Sci Data. 2016 May 24;3:160020. doi: 10.1038/sdata.2016.20 (PMC4878200; doi:10.1038/sdata.2016.20)
Supplement: Supplementary File 2 [file sdata201620-s3.pdf]

Supplementary File 2. Known issues and missing data

**Variable**	**Description**	**Issue**
Missing information		
farmsalev, farmbuyv	sale and purchase value of farm including buildings	Unclear units
gender1-gender8, age1-age8 (or more)	gender and age of household members	Sometimes missing
seas1nam, seas2nam, seas3nam	Name of season 1, 2, 3	Unclear entries e.g. “2-belg”
tib	Time interview began	Missing for Burkina Faso, Ethiopia, Zimbabwe; different time format e.g. hh:mm, hhmm, hh:mmAM, hh:mmPM
tie	Time interview ended	Missing for Burkina Faso, Cameroon, Ethiopia, Zimbabwe; different time format e.g. hh:mm, hhmm, hh:mmAM, hh:mmPM
Several		Missing data indicated by “-99”, “-999”, “.”,” “
Several		Different date format e.g. DD/MM/YY, DD-MM, MM
Several		“Other” not further specified
Value out of range		
fplots	Number of distinct (separated) farm plots	Should be between 1 and 4, 4 for more than 3 plots but other numbers were reported as well in all countries except for Burkina Faso and Zimbabwe
fsystem1, fsystem2	farming system on largest plot and on second largest plot	Should be between 1 and 5 but can be 6 in Ethiopia, Egypt, South Africa, Zambia
livshhmd, livshrmd	Number of days (annual) used by household and hired males in livestock	Number of days larger than 365 in few cases
lvsown	Does household own livestock	Should be 0 (“No”) or 1 (“Yes) but for Cameroon, Ethiopia, Ghana, Kenya, Niger, Senegal, Zimbabwe also 2 is possible which is unclear.
tenure1, tenure2	tenure on largest plot and on second largest plot	Should be between 1 and 7 but can be 8, 12, 15, 16, 20 in Ethiopia, Niger, South Africa; no data for many farms in Kenya
Conflicting information		
landhhm1, landhhf1, landhhc1, landhhm2, landhhf2, landhhc2, landhhm3, landhhf3, landhhc3	Number of household males, females and children who prepare land in season 1, 2, 3	Total number of male adults, female adults and children preparing land larger than household size in few cases

ad731-ad7625	Shifts in long-term temperature and rainfall	Inconsistent responses in few cases e.g. households report to observe changes but also say that they don’t know (multiple entries possible)
rentplot1, rentplot2	Rent paid if plot is leased	Not consistent with tenure type in some cases (rent paid for own land etc.)
